# Intra-amniotic infection: diagnosis, nomenclature, clinical significance, management, and microbiologic tools used for the diagnosis

**DOI:** 10.1128/cmr.00070-26

**Published:** 2026-07-31

**Authors:** Pisut Pongchaikul, Puntabut Warintaksa, Piroon Jenjaroenpun, Anunya Opasawatchai, Rapeewan Settacomkul, Pornpun Vivithanaporn, Suwatcharaporn Hadratchai, Arunee Singsnaeh, Iyarit Thaipisuttikul, Thidathip Wongsurawat, Piya Chaemsaithong

**Affiliations:** 1 Chakri Naruebodindra Medical Institute, Faculty of Medicine, Ramathibodi Hospital, Mahidol University 26687, Samut Prakarn, Thailand; 2 Integrative Computational BioScience Center, Mahidol University 26685, Nakhon Pathom, Thailand; 3 Institute of Infection, Veterinary and Ecological Sciences, University of Liverpool 4591, Liverpool, United Kingdom; 4 Department of Obstetrics and Gynecology, Faculty of Medicine, Ramathibodi Hospital, Mahidol University 26687, Bangkok, Thailand; 5 Division of Medical Bioinformatics, Research Department, Faculty of Medicine, Siriraj Hospital, Mahidol University, Bangkok, Thailand; 6 Siriraj Long-Read Lab (Si-LoL), Faculty of Medicine, Siriraj Hospital, Mahidol University, Bangkok, Thailand; 7 Department of Biomedical Informatics, University of Arkansas for Medical Sciences 12215, Little Rock, Arkansas, USA; 8 Department of Oral Microbiology, Faculty of Dentistry, Mahidol University, Bangkok, Thailand; 9 Department of Pathology, Faculty of Medicine, Ramathibodi Hospital, Mahidol University 26687, Bangkok, Thailand; 10 Department of Microbiology, Faculty of Medicine, Siriraj Hospital, Mahidol University 65106, Bangkok, Thailand; 11 Department of Translational Medicine, Faculty of Medicine, Ramathibodi Hospital, Mahidol University 26687, Bangkok, Thailand; Rush University Medical Center, Chicago, Illinois, USA; Busan Paik Hospital, Inje University College of Medicine, Busan, Republic of Korea

**Keywords:** 16S sequencing, adaptive, adaptive sampling, amniocentesis, amniotic fluid, antibiotic resistance, antibiotics, antimicrobial resistance, bacteria, biomarker, chorioamnionitis, clinical chorioamnionitis, contamination, cultivation, culture, cytokine, delivery, FIRS, fetal inflammatory response syndrome, fever, funisitis, Gram stain, histologic chorioamnionitis, hybrid sequencing, IL-6, infection, inflammation, intra-amniotic inflammation, intra-amniotic infection, interleukin-6, laboratory, long read, matrix metalloproteinase-8, metagenomics, MIAC, microbiology, molecular, molecular microbiologic technique, mycoplasma, nanopore, nanopore sequencing, pathogen, PCR, polymerase chain reaction, premature uterine contractions, preterm delivery, preterm labor, preterm pre-labor rupture of membranes, Sanger, Sanger sequencing, sepsis, sequencing, short read, sterile inflammation, whole genome

## Abstract

Intra-amniotic infection is the main cause of spontaneous preterm birth and adverse maternal–fetal outcomes; therefore, rapid, robust, and accurate diagnosis remains a clinical priority. Conventional microbiological techniques, especially culture-based methods, are limited by long turnaround times and the inability to detect fastidious or unculturable organisms. This review summarizes the diagnosis, nomenclature, clinical significance, management, and laboratory approaches for diagnosing intra-amniotic infection. Targeted nucleic acid amplification methods, including species-specific polymerase chain reaction and broad-range 16S rRNA gene sequencing, have improved the detection of bacterial DNA and enabled the identification of organisms that evade routine culture in intra-amniotic infection. More recently, whole-genome sequencing and metagenomic next-generation sequencing have provided culture-independent strategies for comprehensive pathogen profiling, allowing simultaneous detection of bacteria, viruses, and fungi, as well as characterization of antimicrobial resistance determinants and virulence-associated genes. However, challenges remain, particularly in low-biomass samples such as amniotic fluid, where contamination, host DNA background, and data interpretation can compromise specificity. This review critically evaluates the advantages and limitations of each molecular modality and discusses pre-analytical, analytical, and bioinformatic considerations essential for reliable implementation. Integration of molecular diagnostics into clinical workflows holds promise for improving etiological diagnosis and guiding targeted therapy in intra-amniotic infection, thereby improving maternal and fetal outcomes.

## INTRODUCTION

The conventional paradigm is that the amniotic cavity is sterile of bacteria. There is abundant evidence from cultivation (1–4) and molecular microbiological techniques (5–9) that most pregnancies do not harbor bacteria in the amniotic cavity. The case for viruses has been less well studied; however, the advent of next-generation sequencing should help resolve whether viruses are normally present in the amniotic cavity (7).

Intra-amniotic infection is the most common infection-related diagnosis in labor and delivery units, affecting 9.1% and 24% of preterm intact membranes and preterm pre-labor rupture of membranes, respectively (10, 11). This disorder is one of the leading causes of maternal and neonatal short- and long-term complications. The short-term complications include uterine atony, postpartum hemorrhage, and early-onset neonatal sepsis (4, 12–16). Long-term maternal consequences can also be significant and may include endometritis, infertility, peritonitis, sepsis, adult respiratory distress syndrome, and, rarely, death (4, 13–16). For infants, the long-standing complications are bronchopulmonary dysplasia and cerebral palsy (17, 18). Currently, there is evidence that intra-amniotic infection or inflammation can be treated, and such eradication leads to prolonged pregnancy and improved maternal and neonatal outcomes (19–24). Therefore, early diagnosis is the key to timely management.

The diagnosis of intra-amniotic infection requires laboratory analysis; the ideal laboratory test should be rapid and accurate to support proper management, e.g., timing delivery or targeted, early administration of antimicrobial agents. Advances in microbiology laboratory techniques have evolved over time. In the past, cultivation remained the gold standard for diagnosing infections (25–28). Currently, molecular microbiological techniques are gaining momentum for pathogen identification (29–33). In contrast to “intra-amniotic infection,” the term “clinical chorioamnionitis” is typically diagnosed based on clinical criteria or signs, which have been proven to be inaccurate for diagnosing intra-amniotic infection (16, 34, 35).

Another important issue is an emerging antibiotic-resistant pathogen, which is a serious global threat that has adversely affected both clinical and therapeutic outcomes, with consequences ranging from treatment failures to antimicrobial resistance and the need for safer alternative drugs (36–38). Hence, individualized, targeted antimicrobial treatment for intra-amniotic infection should be considered based on the causative pathogen and its antimicrobial resistance profile. For the purposes of this review, which focuses on criteria for diagnosis, nomenclature, clinical significance, clinical management, microbiology, and the microbiologic laboratory tools used to diagnose intra-amniotic infection, these topics are retained to elucidate this condition.

## DIAGNOSIS AND NOMENCLATURE OF INTRA-AMNIOTIC INFECTION

### Diagnosis

Accurate diagnosis of intra-amniotic inflammation and intra-amniotic infection requires the analysis of amniotic fluid. Under normal circumstances, amniotic fluid does not contain bacteria or white blood cells. The diagnostic investigation and laboratory analysis of intra-amniotic infection are similar to those of meningitis, which requires cerebrospinal fluid obtained by lumbar puncture. For the diagnosis of intra-amniotic infection, therefore, amniotic fluid retrieved by transabdominal amniocentesis is necessary. Subsequently, tests for the diagnosis of intra-amniotic inflammation (white blood cell count, glucose concentration, interleukin-6 [IL-6], or matrix metalloproteinase-8 [MMP-8]) and microorganisms in the amniotic cavity (culture or molecular tests) are performed. Thus far, it is now clear that maternal symptoms and signs are insensitive indicators of intra-amniotic infection (34, 39), and therefore, transabdominal amniocentesis is currently required to diagnose this condition. Previous studies have demonstrated the value of non-invasive methods, such as maternal or cervicovaginal fluid biomarkers, for the identification of intra-amniotic infection; however, these methods are not yet ready for clinical use and require further clinical validation (40–42).

### Nomenclature

**Fig 1:**
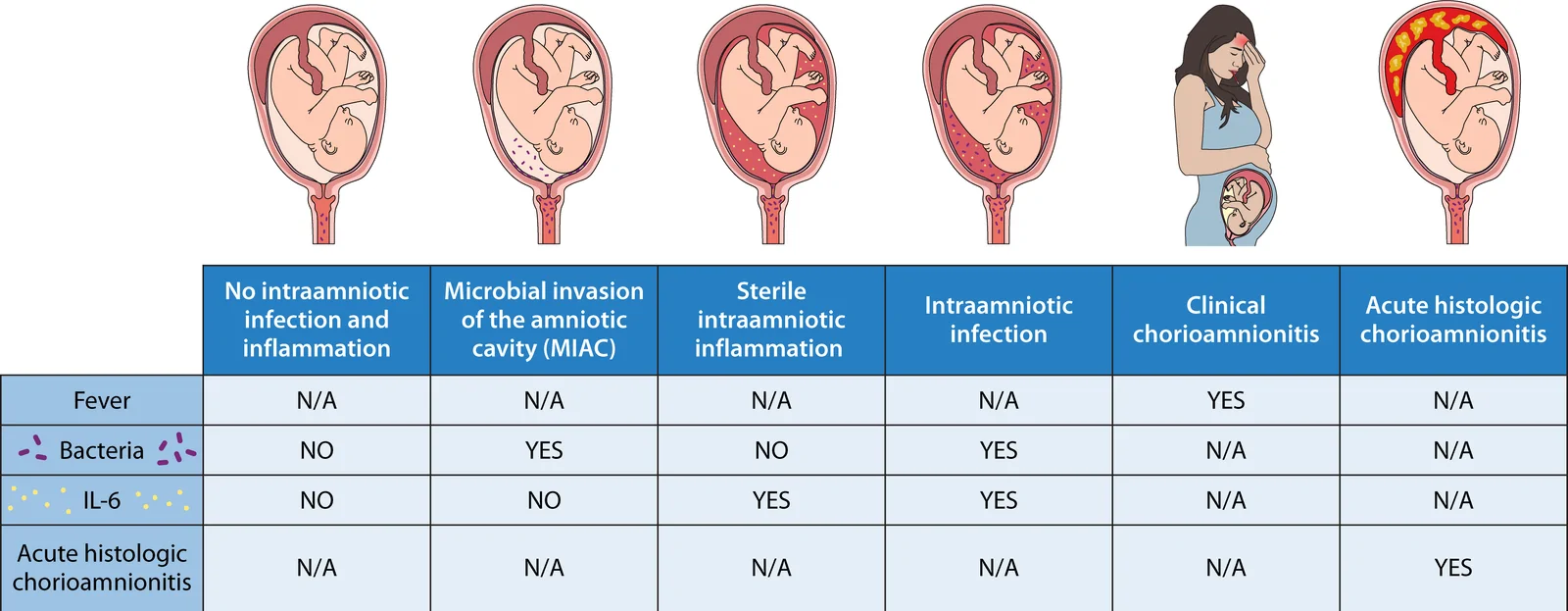
Nomenclature of intra-amniotic infection and associated conditions. Microbial invasion of the amniotic cavity, intra-amniotic inflammation, sterile intra-amniotic inflammation, and intra-amniotic infection. Microbial invasion of the amniotic cavity is characterized by the presence of microorganisms in amniotic fluid obtained by transabdominal amniocentesis, as detected by cultivation methods and/or molecular microbiological techniques. Intra-amniotic inflammation is defined by the presence of inflammatory cells (white blood cell count ≥50 cells/mm^3^) or an elevated level of a biomarker of inflammation, IL-6 concentration ≥2.6 ng/mL, or matrix MMP-8 concentration greater than 23 ng/mL. Sterile intra-amniotic inflammation is defined as the presence of intra-amniotic inflammation in the absence of microorganisms. When intra-amniotic inflammation is accompanied by microorganisms in the amniotic cavity, the condition is referred to as intra-amniotic infection. N/A, not available.

Often, the terms “intra-amniotic infection,” “clinical chorioamnionitis,” “acute histologic chorioamnionitis,” “microbial invasion of the amniotic cavity (MIAC),” and “triple I” are used interchangeably. Yet, they are not equated or identical. The following are the diagnostic criteria for each condition (Table 1 and Fig. 1).

**TABLE 1:** Terminology

Conditions	Definitions
Microbial invasion of the amniotic cavity	The presence of organisms in the amniotic fluid retrieved by transabdominal amniocentesis and detected by cultivation methods and/or molecular microbiological techniques.
Intra-amniotic inflammation	An increased number of inflammatory cells (white blood cells) or cytokines (interleukin-6) or matrix metalloproteinase 8 in amniotic fluid. • White blood cell count ≥50 cells/mm^3^, or • Amniotic fluid IL-6 concentration ≥2.6 ng/mL, or • Amniotic fluid MMP-8 concentration >23 ng/mL
Intra-amniotic infection	The combination of microbial invasion of the amniotic cavity and intra-amniotic inflammation.
Sterile intra-amniotic inflammation	The presence of intra-amniotic inflammation with the absence of microorganisms detected by both cultivation methods and molecular microbiological techniques.
Clinical chorioamnionitis	This condition is diagnosed by the clinical criteria: maternal fever (temperature 37.8°C or ≥38.0°C) and the combination of at least two of the following clinical signs: (i) maternal tachycardia (heart rate >100 beats/min); (ii) fetal tachycardia (heart rate >160 beats/min); (iii) uterine tenderness; (iv) purulent or foul-smelling amniotic fluid or vaginal discharge; and (v) maternal leukocytosis (white blood cell count >15,000/mm^3^).
Acute histologic chorioamnionitis	This condition is a histopathological diagnosis of the placenta, characterized by the presence of neutrophils in the chorioamniotic membranes or the chorionic plate. This condition represents a maternal inflammatory response.
Acute funisitis	This condition is a histopathological diagnosis of the placenta, characterized by inflammation of the umbilical cord (umbilical vein, umbilical artery, and Wharton’s jelly). This condition represents a fetal, not a maternal, inflammatory response.

**Fig 2:**
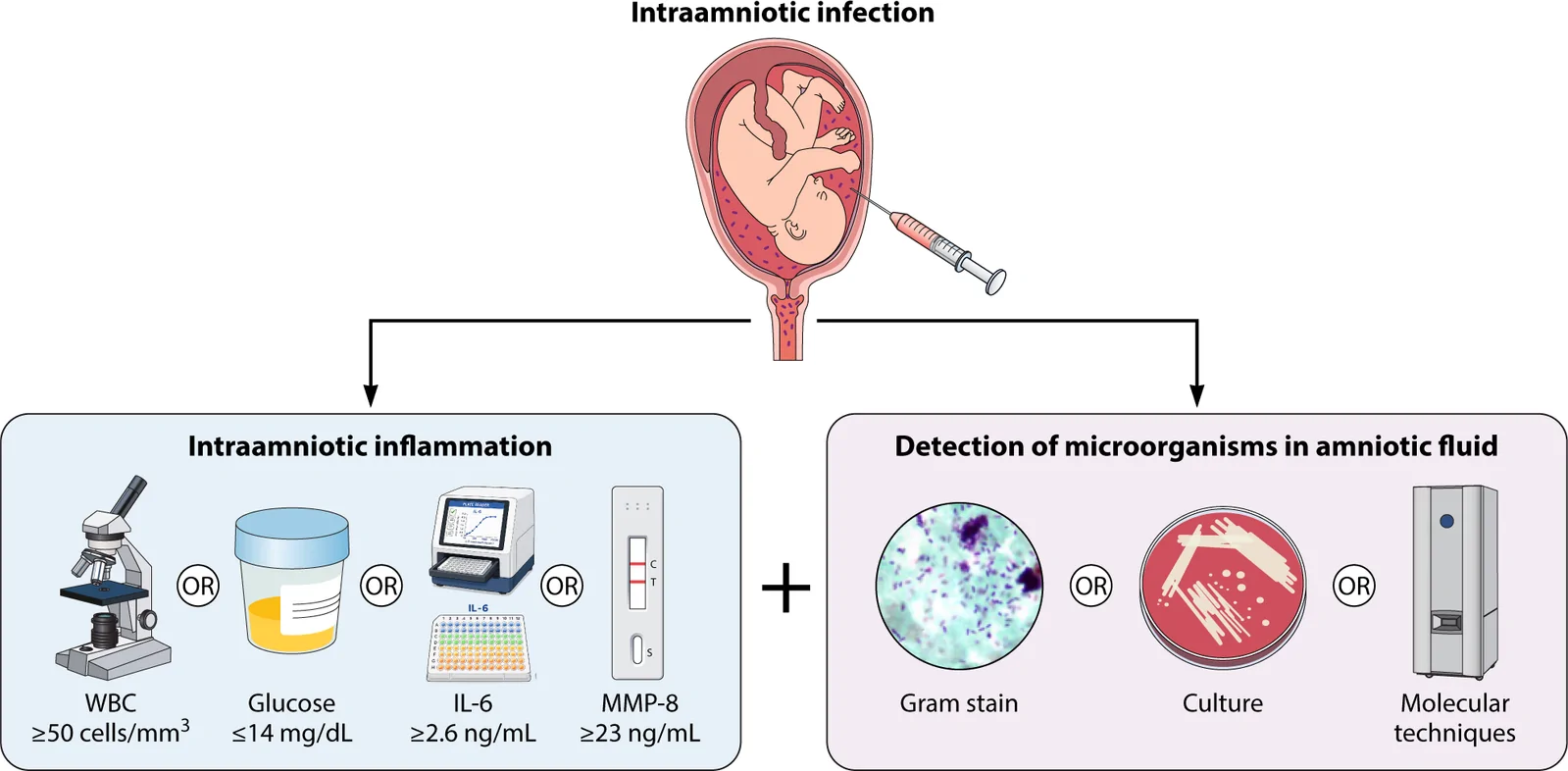
Criteria for the diagnosis of intra-amniotic infection. WBC, white blood cell.

- Microbial invasion of the amniotic cavity is defined as the presence of microorganisms in amniotic fluid retrieved by transabdominal amniocentesis that can be detected by cultivation methods or molecular microbiological techniques without intra-amniotic inflammation. The clinical significance of MIAC is uncertain, as most cases without evidence of intra-amniotic inflammation are likely due to contamination by skin flora, laboratory bacteria, or reagents (43, 44).
- Intra-amniotic inflammation is characterized by the presence of an inflammatory response in the amniotic cavity, which can be detected by the presence of inflammatory cells, for instance, white blood cell count (>50 cells/mm^3^), or an elevation of a pro-inflammatory mediator concentration in amniotic fluid, such as IL-6 (≥2.6 ng/mL) (34), or MMP-8 (≥23 ng/mL) (45–47).
- Sterile intra-amniotic inflammation is defined as a condition in which there is intra-amniotic inflammation with the absence of detectable microorganisms. This term should be reserved for samples that are negative for microorganisms detected by both cultivation methods and molecular microbiological techniques (48, 49). Sterile intra-amniotic inflammation has been attributed to danger signals (or alarmins) that are released during cellular stress or cell necrosis (50, 51).
- Intra-amniotic infection is characterized by the presence of a microorganism detected objectively by amniotic fluid culture, Gram stain, or molecular microbiology techniques, coupled with intra-amniotic inflammation; therefore, this term is primarily diagnosed by “laboratory criteria” for amniotic fluid analysis (Fig. 2) (16). In some cases, such as patients with premature rupture of membranes and oligo- or anhydramnios, the diagnosis can be difficult as analysis of amniotic fluid is not possible. Hence, the presence of microorganisms in the amniotic cavity together with acute inflammatory lesions of the placenta, such as acute histologic chorioamnionitis or acute funisitis, which is reflective of intra-amniotic infection/inflammation, is sufficient (52, 53).
- Clinical chorioamnionitis is a clinical syndrome or clinical diagnosis defined as the following clinical criteria: maternal fever (temperature ≥37.8°C or ≥38.0°C) and two of the following signs or symptoms: uterine tenderness, maternal tachycardia (>100 beats per min), maternal leukocytosis (>15,000 cells/m^3^), fetal tachycardia (>160 beats per min), and purulent or foul-smelling amniotic fluid (12, 39, 54, 55). Importantly, this condition is diagnosed by “clinical criteria or diagnosis,” which is different from “intra-amniotic infection,” which is diagnosed using “laboratory criteria.”
- Triple I is a term referring to “intra-amniotic infection or intra-amniotic inflammation or both,” yet they are not the same. This term was proposed by a National Institute of Child Health and Human Development expert panel to replace the term “chorioamnionitis,” a clinical diagnosis (56). However, the term intra-amniotic infection or inflammation requires examination of amniotic fluid, which is a laboratory diagnosis, not a clinical diagnosis, to determine whether the most likely cause is microbial or sterile (57).

## EPIDEMIOLOGY AND FREQUENCY OF INTRA-AMNIOTIC INFECTION

A fetus is considered living in a sterile environment since the frequency of MIAC is approximately <1% by the use of cultivation and molecular microbiological techniques (8, 58). In addition, a sterile environment has been maintained until term gestation, as <1% of amniotic fluid of women not in labor at term contains bacteria (3, 7). These findings are confirmed by both cultivation and molecular microbiological techniques. Hence, the detection of bacteria in the amniotic cavity is a pathologic finding. The frequency of intra-amniotic infection depends on clinical diagnosis (i.e., preterm labor with intact membranes, preterm pre-labor rupture of membranes, cervical insufficiency, or clinical chorioamnionitis), gestational age at presentation, and the methods used to detect microorganisms.

**Fig 3:**
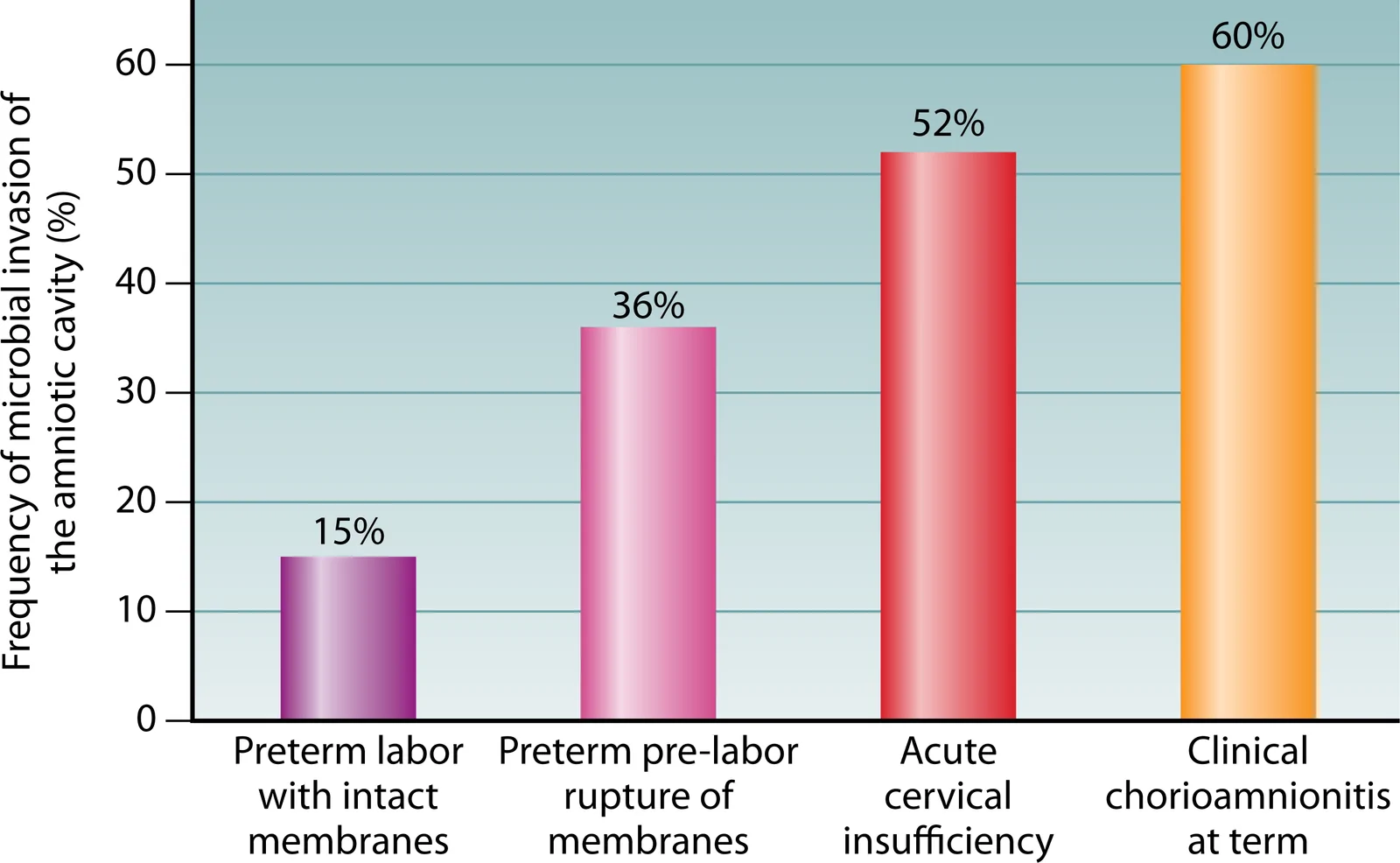
The frequencies of microbial invasion in the amniotic cavity in each obstetrical syndrome.

In preterm labor with intact membranes, approximately 10% of patients have microorganisms detected in amniotic fluid by cultivation (59–62). The number increases to 15% when molecular microbiological techniques are used (10). In patients with preterm pre-labor rupture of membranes, the rate of amniotic fluid cultures positive for microorganisms is approximately 32.4% (63). However, the frequency increases to 50% if a molecular microbiological test is employed (11). In patients with cervical insufficiency, a condition defined as the inability of the uterine cervix to retain a pregnancy in the absence of uterine contractions, the frequency of intra-amniotic infection ranged from 8% to 52% with the use of cultivation combined with a molecular test (43, 64–68). This is important as the treatment with cervical cerclage in patients with pre-existing intra-amniotic infection is associated with adverse pregnancy outcomes and maternal sepsis (69–71). In clinical chorioamnionitis at term, defined by intrapartum fever plus systemic maternal clinical signs, 60% of patients have proven intra-amniotic infection, 24% have sterile intra-amniotic inflammation, and 15% have no evidence of intra-amniotic infection or inflammation (39, 72, 73). Therefore, intrapartum fever does not necessarily indicate the presence of intra-amniotic infection. Importantly, a majority of patients with intra-amniotic infection have subclinical infection, as the patients do not have fever. Figure 3 demonstrates the frequency of intra-amniotic infection in each clinical syndrome.

Among organisms causing intra-amniotic infection, the most common are *Ureaplasma* species (*Ureaplasma parvum* and *Ureaplasma urealyticum*), *Fusobacterium* spp., and *Mycoplasma hominis* (10, 11, 25, 26, 39, 43, 44, 72, 74–79), and polymicrobial infections occur in approximately 70% of intra-amniotic infections (16). The frequency of intra-amniotic infection is similar worldwide (20–22, 48, 80–87).

## CLINICAL RISK FACTORS OF INTRA-AMNIOTIC INFECTION

Clinical risk factors for intra-amniotic infection include gestational age at clinical presentation, amniotic fluid index, clinical diagnosis (preterm, preterm pre-labor rupture of membranes, cervical insufficiency, or clinical chorioamnionitis), cervical length, or sonographic sign of cervical sludge. Specifically, the earlier the gestational age at preterm birth, the more likely it is to have microbial invasion of the amniotic cavity, which is associated with respiratory distress syndrome, bronchopulmonary dysplasia, and neonatal death (88). Patients with preterm labor and intact membranes whose gestational age was 23–26 weeks had 45% positive amniotic fluid cultures, while those with gestational age 31–34 weeks had 11.4% positive amniotic fluid cultures (88).

In patients with preterm pre-labor rupture of membranes, the rate of intra-amniotic infection is inversely related to amniotic fluid index (89, 90). The rate of culture positivity in the amniotic fluid is as high as 79% in patients with an amniotic fluid index <5 cm compared to 30% in those with a normal amniotic fluid index (>5 cm) (90). Moreover, patients with preterm pre-labor rupture of membranes and signs of labor have a significantly higher rate of positive culture than those without labor (39% vs 25%), and this rate increases to 75% with the onset of preterm labor (91). Such observation underscores that the onset of preterm labor in patients with preterm pre-labor rupture of membranes is most likely to reflect the presence of intra-amniotic infection.

**Fig 4:**
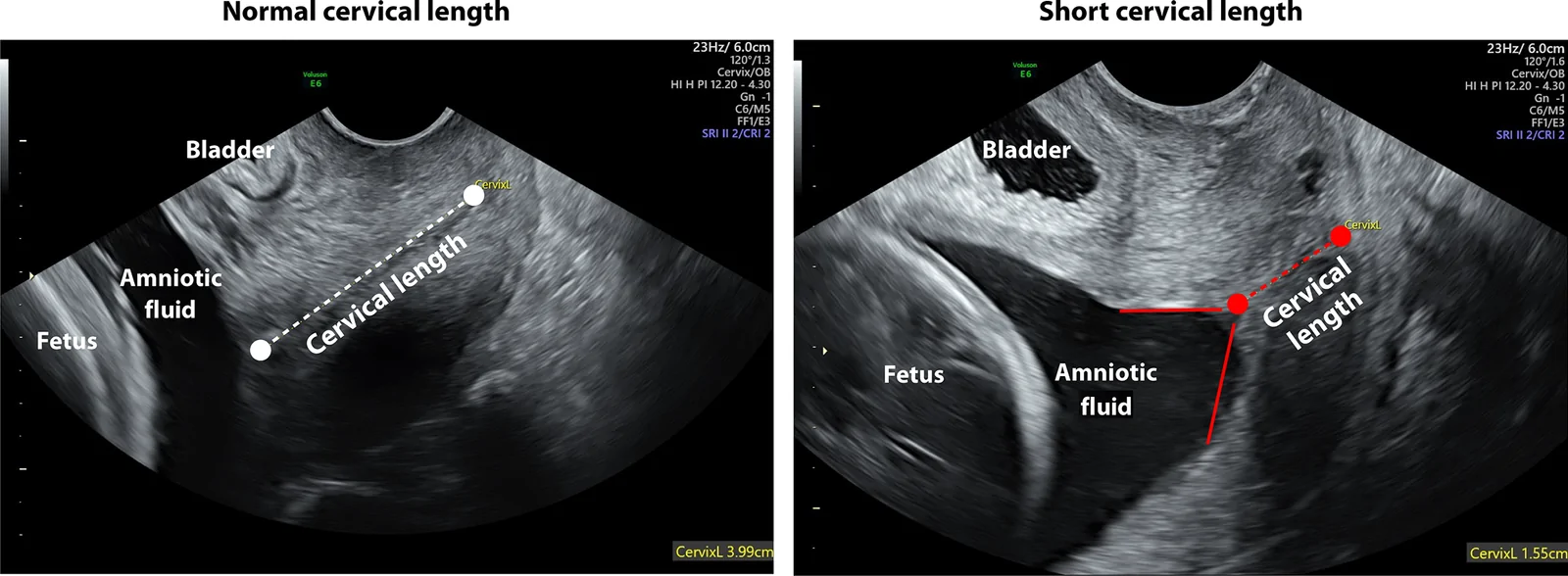
A transvaginal ultrasound demonstrates the measurement of cervical length. A normal cervical length (≥2.5 cm) with a T-shape cervical length (left). A short cervix shows cervical length of <2.5 cm with Y-shaped funneling (right).

**Fig 5:**
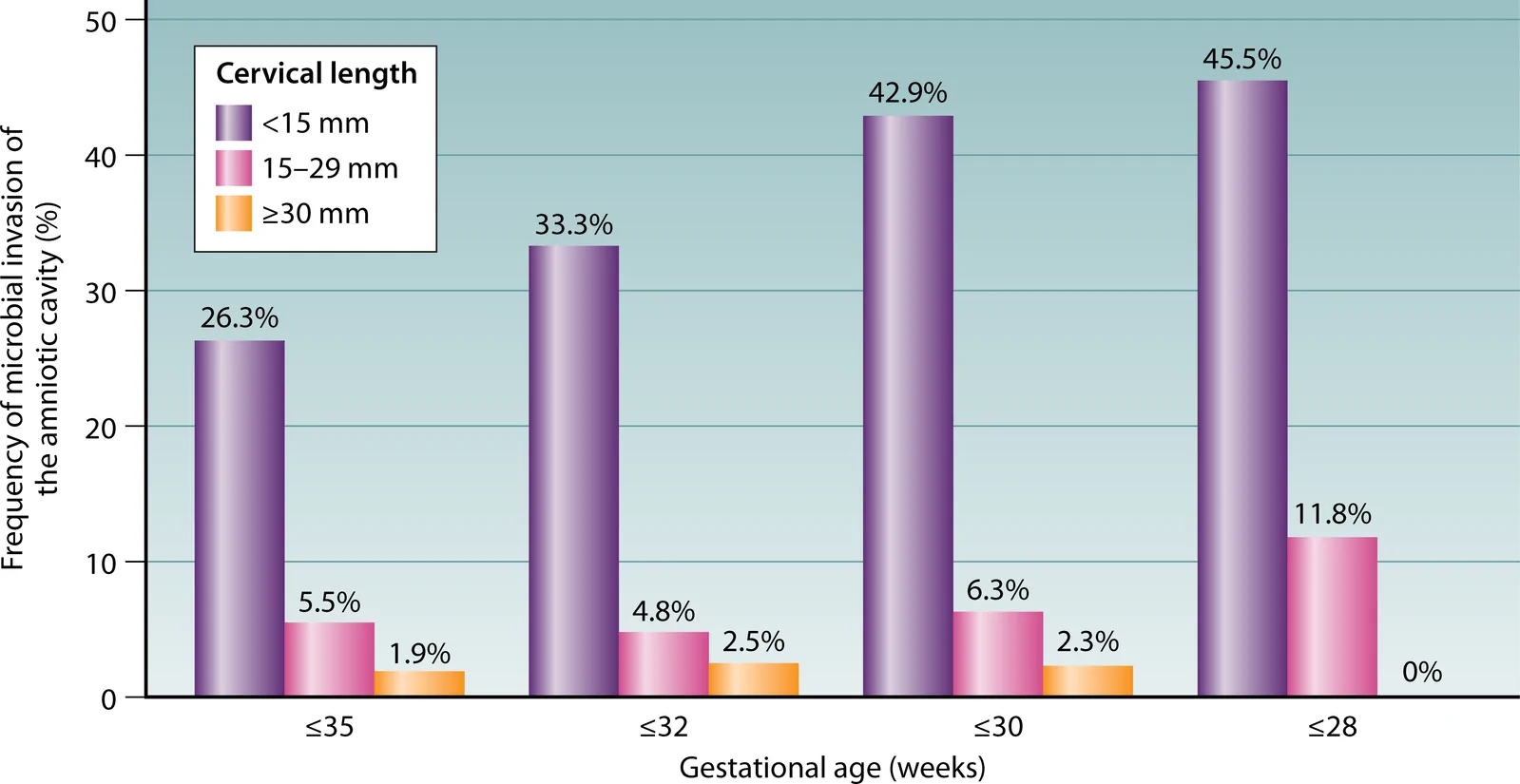
The relationship between the frequencies of microbial invasion in the amniotic cavity and ultrasonographic cervical length. Data from reference 102.

**Fig 6:**
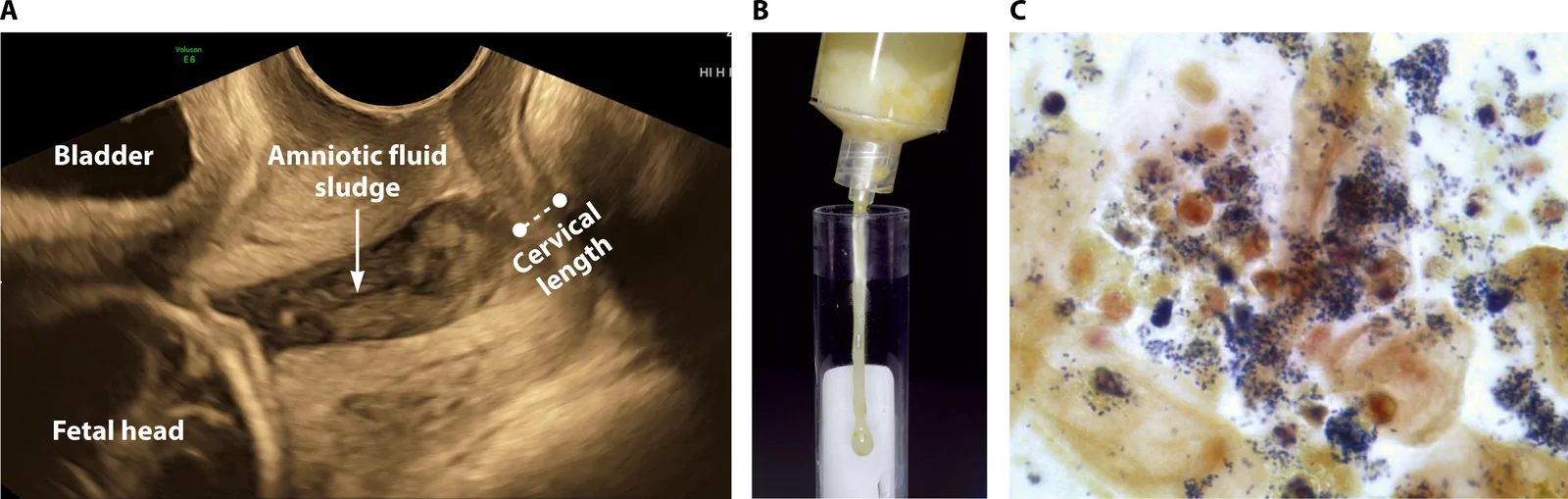
Amniotic fluid sludge. (**A**) Two-dimensional ultrasound image showing amniotic fluid sludge (particulate matter floating above the cervix; white arrow) in a patient with a short cervix. (**B**) Photograph showing the appearance of amniotic fluid “sludge,” aspirated during a transvaginal needle amniotomy under transabdominal ultrasound guidance. (**C**) Image demonstrating the presence of epithelial cells and many gram-positive cocci; neutrophils are also evident. Panel **A** image courtesy of Dr. Piya Chaemsaithong. Panel **B and C** images republished from reference 97 with permission of the publisher.

A sonographic short cervix in the second trimester, defined as a cervical length of 25 mm or less, is a powerful predictor of spontaneous preterm delivery (92, 93) (Fig. 4). The shorter the cervical length, the higher the risk of microbial invasion of the amniotic cavity (Fig. 5). Subclinical microbial invasion of the amniotic cavity was detected in 9% of asymptomatic patients with a sonographically short cervix (49, 94). The presence of “amniotic fluid sludge” in these patients has been shown to be evidence of intra-amniotic infection and inflammation, impending preterm delivery, and chorioamnionitis (95–99). The term “amniotic fluid sludge” refers to the presence of echogenic, free-floating aggregates of particulate matter within the amniotic cavity and often near the internal cervical os in women with intact membranes (Fig. 6) (97). Microorganisms such as *Ureaplasma urealyticum, Mycoplasma hominis, Streptococcus mutans, and Candida albican*s have been cultured from aspirated samples of amniotic fluid sludge (98). Scanning electron microscopy of amniotic fluid sludge revealed bacteria organized into biofilms. Indeed, immunohistochemistry with wheat germ agglutinin revealed the presence of matrix material that forms the biofilm’s structural framework (95–98, 100, 101). The presence of biofilms has an important clinical implication, as these structures are typically resistant to antibiotic treatment.

## PATHOGENESIS, ROUTE, AND STAGES OF INTRA-AMNIOTIC INFECTION

The dominant route by which microorganisms gain access to the amniotic cavity is ascending migration from the lower genital tract, progressing sequentially from the vagina and cervix through the decidua, chorioamniotic membranes, amniotic fluid, and ultimately to the fetus itself (2, 4, 103). For decades, the ascending pathway was inferred from indirect microbiological evidence, matching organisms recovered from the vagina and amniotic fluid by conventional culture (103, 104). Whole-genome hybrid sequencing demonstrated that microorganisms recovered from four distinct clinical compartments, including the vaginal ecosystem, amniotic fluid, chorioamniotic membranes, and neonatal blood, in a case of Group B streptococcus (GBS) intra-amniotic infection and early-onset neonatal sepsis were genomically identical, sharing the same sequence type (ST1), clonal complex (CC1), and serotype (Ib), with highly similar DNA sequences across all four isolates (23). This study provides the first genomic-level evidence that the ascending pathway is the mechanism underlying intra-amniotic infection and directly leads to neonatal bacteremia.

A second, less common route of intra-amniotic infection is hematogenous dissemination from the maternal bloodstream. This pathway is principally implicated in *Listeria monocytogenes* intra-amniotic infection, in which transplacental spread from a bacteremic mother to the fetus, followed by amniotic cavity invasion, occurs without prior colonization of the lower genital tract. Molecular evidence was obtained through a comprehensive microbiological and genomic workup in a patient with spontaneous preterm labor. *L. monocytogenes* was detected in amniotic fluid and chorioamniotic membranes but was absent from the vaginal ecosystem. Whole-genome hybrid sequencing identified both isolates as ST1, clonal complex CC1, and serotype 4b (105). Placental pathology revealed extensive acute intervillositis with intracellular, rod-shaped bacteria in the intervillous space, confirmed by Brown-Hopps and Warthin-Starry staining and colorimetric *in situ* hybridization. The detection of *L. monocytogenes* in the intervillous space, representing maternal circulation, in the absence of vaginal involvement, provided definitive evidence that hematogenous transplacental transmission, rather than ascending infection, was the operative route in this case (105). Altogether, through the use of genomic case studies, a dual-route model of intra-amniotic infection has been established, now supported by organism-level molecular evidence.

Pregnancy remodels maternal immunity, altering susceptibility to both ascending and hematogenous infections. The gestational immune environment shifts progressively toward an anti-inflammatory phenotype to prevent rejection of the semi-allogeneic fetus, involving changes in decidual natural killer cell function, expansion of regulatory T cells, and attenuation of Th1 effector responses (106–110). While essential for implantation and fetal tolerance, these adaptations reduce mucosal barrier defense in the lower genital tract and increase susceptibility to intracellular pathogens, such as *Listeria*, which exploits gestational immunological suppression particularly effectively.

**Fig 7:**
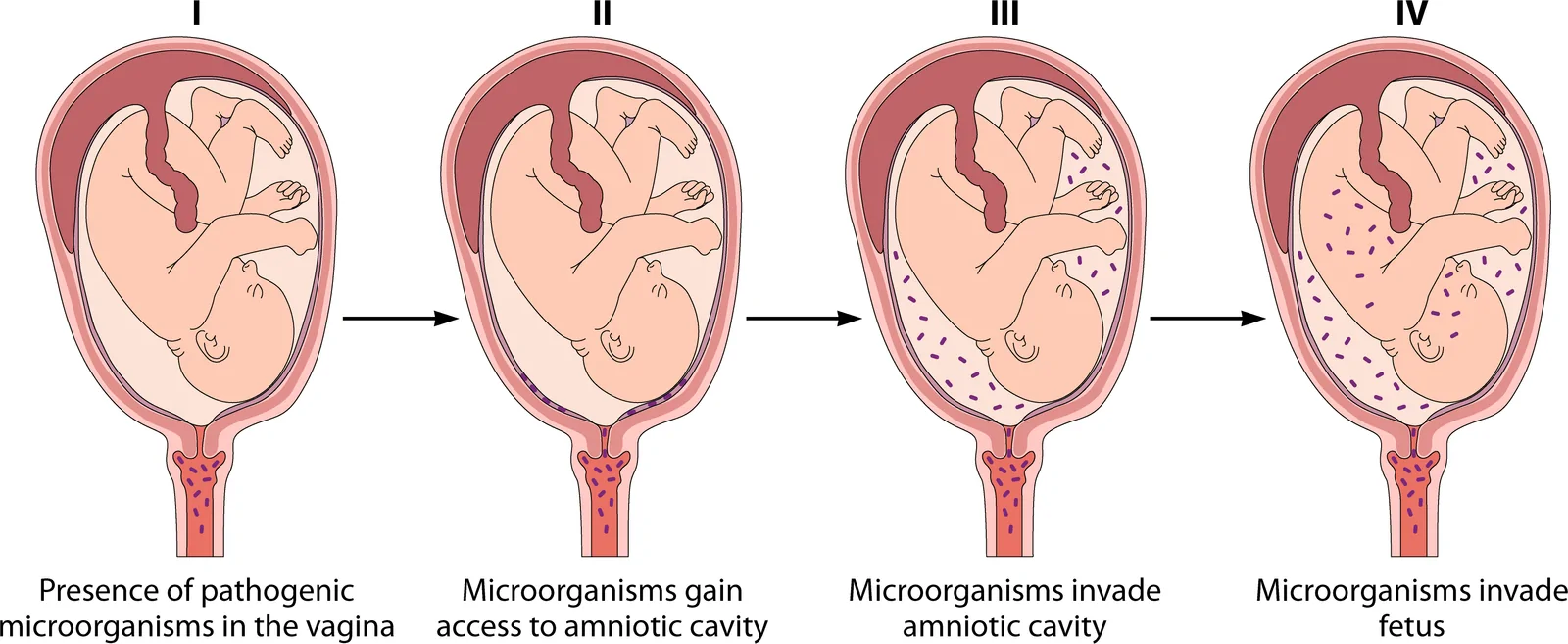
The stages of ascending infection. The first stage corresponds to a change in the vaginal/cervical microbial flora or the presence of pathogenic organisms in the cervix. Once microorganisms gain access to the amniotic cavity, they reside in the lower uterine pole between the membranes and the chorion (stage 2). The microorganisms may proceed through the amnion (amnionitis) into the amniotic cavity, leading to an intra-amniotic infection (stage 3). The microorganisms may invade the fetus through different ports of entry (stage 4) (modified from reference 2 with permission from the publisher).

When microbial invasion of the amniotic cavity occurs, innate immune signaling, driven by toll-like receptor activation, nuclear factor-κB, and the downstream release of interleukin-1β, interleukin-6, tumor necrosis factor-α, and matrix metalloproteinases, triggers prostaglandin-mediated uterine contractions and cervical remodeling, mechanistically linking infection to preterm labor (108, 111, 112). The fetal inflammatory response syndrome, characterized by systemic fetal inflammation with umbilical cord IL-6 elevation ≥11 pg/mL, represents the fetal counterpart of this process and is associated with the worst perinatal outcomes, including bronchopulmonary dysplasia, periventricular leukomalacia, and sepsis (15). Figure 7 shows the stages of intra-amniotic infection. The first stage corresponds to a change in the vaginal/cervical microbial flora or the presence of pathogenic organisms in the cervix. Once microorganisms gain access to the amniotic cavity, they reside in the lower uterine pole between the membranes and the chorion (stage 2). The microorganisms may proceed through the amnion (amnionitis) into the amniotic cavity, leading to an intra-amniotic infection (stage 3). The microorganisms may invade the fetus through different ports of entry (stage 4).

## MICROBIOLOGY OF INTRA-AMNIOTIC INFECTION

The microorganisms found in the amniotic cavity during intra-amniotic infection are typically derived from ascending vaginal microbiota. The most common are genital *Mycoplasma* species, particularly *Ureaplasma urealyticum*. However, other microorganisms, including *Mycoplasma hominis, Streptococcus agalactiae, Escherichia coli,* anaerobic bacteria such as *Fusobacterium* species*,* and *Gardnerella vaginalis*, are also present. These bacteria are predominantly fastidious, making cultivation difficult. Important but relatively less common pathogens include *Listeria monocytogenes* and *Treponema pallidum* subsp. *pallidum*, which are transmitted via the maternal bloodstream. Table 2 demonstrates common microorganisms found in the amniotic cavity (16). In approximately 50% of patients with intra-amniotic infection, more than one microorganism is isolated from amniotic fluid. The inoculum size differs considerably, and in 71% of cases, more than 10^5^ colony-forming units per milliliter are found (25). *Chlamydia trachomatis* is rarely found in the amniotic fluid (113).

**TABLE 2:** A list of common microorganisms detected in the amniotic fluid in patients with intra-amniotic infection

Microorganisms identified in amniotic fluid
*Ureaplasma* species (*Ureaplasma urealyticum* and *Ureaplasma parvum*)
*Streptococcus agalactiae*
*Gardnerella vaginalis*
*Lactobacillus* species
*Bacteroides* species
*Fusobacterium* species (*Fusobacterium nucleatum* and *Fusobacterium varium*)
*Sneathia* species
*Streptococcus viridans*
*Porphyromonas* species
*Veillonella* species
*Peptostreptococcus* species
*Escherichia coli*
*Pseudomonas aeruginosa*
*Staphylococcus aureus*
*Candida* species
*Enterococcus faecalis*
*Acinetobacter* species
Others: *Candida albicans, Staphylococcus epidermidis,* and *Propionibacterium acnes*

Characterization of viral invasion of the amniotic cavity is less well reported than that of bacterial invasion. Indeed, most studies have employed targeted polymerase chain reaction (PCR) approaches for common viruses, such as human herpes virus 6, cytomegalovirus, parvovirus B19, Epstein-Barr virus, etc. (114–116). The frequency of virus invasion of the amniotic cavity is 2.2% (16 of 729) in pregnant women undergoing second-trimester amniocentesis for genetic indications (117). The frequency of the virus in amniotic fluid in patients with preterm pre-labor rupture of membranes is extremely rare (115, 116). Characterization of the amniotic fluid virome is a research frontier. *Candida* intra-amniotic infection has also been reported (118–122). Clinical risk factors for intra-amniotic *Candida* infection include the presence of an intrauterine device and pregnancy after *in vitro* fertilization (119, 120, 123–126). *Candida* intra-amniotic fluid infection is not the subject of this review.

## MANAGEMENT OF INTRA-AMNIOTIC INFECTION

Proper management of intra-amniotic infection during pregnancy requires rapid and accurate diagnosis. Thus far, maternal signs and symptoms have not been sensitive or accurate for diagnosing intra-amniotic infection; therefore, the gold standard for diagnosing and managing preterm labor syndromes remains analysis of amniotic fluid by transabdominal amniocentesis (34, 127). Evidence supports one recent study that compared two strategies for the management of patients with pre-labor rupture of membranes (*n* = 513) on pregnancy outcomes, including latency period, chorioamnionitis, or maternal and neonatal adverse outcomes (83). The two strategies were (i) individualized management based on amniocentesis result (*n* = 189); and (ii) standard management with standard antibiotic regimen and routine surveillance (*n* = 314). Individualized management includes administering ampicillin, gentamicin, and azithromycin, as well as targeted therapy based on microbiology results (e.g., changing the antibiotic regimen or discontinuing antibiotic therapy). The authors concluded that the individualized management group had a higher frequency of latency period >7 days (76.0% vs 41.6%, *P* < 0.001) and longer duration from latency to birth (18.1 ± 14.7 days vs 9.7 ± 9.7 days, *P* < 0.001) but lower rates of preterm birth <32 weeks (72.1% vs 90.5%, *P* < 0.001), neonatal intensive care unit admission (75.6% vs 83.0%, *P* = 0.046), and neonatal respiratory support at 28 days of life (16.1% vs 26.1%, *P* < 0.01) compared with the standard management group (83). Although there was a higher rate of clinical chorioamnionitis in the individualized treatment group (34.5% vs 22.0%, *P* < 0.01), no differences in histological chorioamnionitis, funisitis, or composite infectious maternal outcomes were observed. These results support the management of pre-labor rupture of membranes based on the amniocentesis result (83). In addition, an observational study has determined the antibiotic exposure duration between the management based on amniocentesis results and the standard management in patients with pre-labor rupture of membranes (128). The standard management group receives a broad-spectrum antibiotic regimen (including intravenous ampicillin and gentamicin, and a single dose of oral azithromycin, or intravenous ceftriaxone and clarithromycin) for at least 5 days. In contrast, the amniocentesis management group receives antibiotic treatment based on microbiological results for 7–10 days. In patients without intra-amniotic infection, the antibiotic treatment was discontinued at 48 h (128). The authors reported that management based on amniocentesis results has shorter antibiotic exposure duration (median [1st quartile–3rd quartile], 2 [2–3] days vs 5 [4–5] days; *P* < 0.0001) and shorter hospitalization duration (median [1st quartile–3rd quartile], 5 [4–9] days vs 11 [5–21] days; *P* = 0.001) compared to the standard management group (128). Altogether, evidence has supported the role of amniocentesis-based management in preterm labor syndrome.

Once intra-amniotic infection is diagnosed, gestational age at the diagnosis and fetal health status are the primary parameters for clinical management decisions. For example, when intra-amniotic infection occurs close to term, termination of pregnancy is recommended in order to obtain a favorable outcome for both mother and neonate. However, management of intra-amniotic infection that occurs in preterm gestations varies, ranging from antimicrobial treatment with careful monitoring to delivery in some cases.

### Antimicrobial regimen to treat intraamniotic infection

The current standard antimicrobial treatment in preterm labor is the administration of ampicillin. Indeed, ampicillin has historically been the main empiric coverage for *L. monocytogenes* and GBS, both of which retain susceptibility to ampicillin. When combined with gentamicin, ampicillin provides standard empiric coverage for suspected intra-amniotic infection (12). However, ampicillin resistance among *E. coli* isolates, which account for a meaningful proportion of gram-negative intra-amniotic infections, has increased substantially in recent decades.

In obstetrics, *E. coli* isolates from pregnant women have also shown ampicillin resistance rates exceeding 65% in several regional studies, and dual ampicillin-gentamicin resistance is increasingly reported in settings with high prevalence of extended-spectrum beta-lactamase-producing Enterobacteriales (129). In addition, it is notable that ampicillin, which is given during intrapartum for Group B *Streptococcus* prophylaxis in patients with preterm labor, does not provide coverage for *Ureaplasma* and *Mycoplasma* species, the most frequent microorganisms in intra-amniotic infection (130). Such species lack a cell wall and are resistant to beta-lactams and glycopeptides (131). Gentamicin is also ineffective (132). Therefore, proper and effective antimicrobial treatment is needed.

In nonhuman primates, azithromycin eradicates *Ureaplasma* from the amniotic cavity and reduces fetal lung injury (133, 134). Clarithromycin, with superior transplacental passage, is effective, shows lower resistance rates (0.9%–25%) (133, 134), and reduces *Ureaplasma* DNA burden in patients with preterm pre-labor rupture of membranes (22).

In humans, previous reports demonstrated that the antibiotic regimen consisting of ceftriaxone, metronidazole, and clarithromycin (also called “triple antibiotics”) was effective in treating proven intra-amniotic infection and intra-amniotic inflammation in patients presenting with preterm labor with intact membranes (21), preterm pre-labor rupture of membranes (19, 135), acute cervical insufficiency (20, 136), or mid‐trimester miscarriage (137). In preterm labor with intact membranes, 50 patients with intra-amniotic infection and/or intra-amniotic inflammation (determined by cultivation and polymerase chain reaction for *Ureaplasma* spp. and IL-6 concentrations) received triple antibiotics for 14 days. Amniocentesis was repeated 1 week after antibiotic treatment to assess treatment response and the presence of intra-amniotic infection/inflammation. Resolution of intra-amniotic infection and/or intra-amniotic inflammation was demonstrated in 79% (15/19) of patients. Overall treatment success, defined as resolution of intra-amniotic infection/intra-amniotic inflammation or delivery at 37 weeks or more, was 84% (16/19) (21).

In conclusion, empiric regimens, including a triple-antibiotic regimen, should be administered to patients with proven intraamniotic infection or inflammation. Subsequently, the targeted antibiotic regimen should be guided by local antimicrobial resistance surveillance data, and genomically informed therapy should be implemented as rapidly as microbiological data allow.

### Antimicrobial resistance and organism-directed therapy

The continued appropriateness of clarithromycin in this regimen warrants comment, given rising macrolide resistance among genital mycoplasmas. A systematic review and meta-analysis of 57 studies (8,966 samples from 21 countries) demonstrated that macrolide resistance-associated mutations in *Mycoplasma genitalium* rose from approximately 10% before 2010 to over 51% by 2016–2017, with the highest prevalence in the Western Pacific and Americas (138). Remarkably, azithromycin-resistant *Ureaplasma urealyticum* and *Mycoplasma hominis* are also increasingly documented among pregnant women, and azithromycin-resistant cervical mycoplasma infection is associated with significantly higher rates of adverse outcomes, including preterm birth, preterm pre-labor rupture of membranes, and stillbirth (139). Clarithromycin and azithromycin share the same 23S rRNA target; macrolide resistance-associated mutations at positions A2058 and A2059, therefore, confer cross-resistance to both agents (138).

This has two practical consequences for the management of intra-amniotic infection. First, the empiric triple-antibiotic regimen remains a reasonable choice when *Mycoplasma* or *Ureaplasma* infection is suspected and macrolide resistance testing is unavailable, because the ceftriaxone and metronidazole components provide broad gram-negative and anaerobic coverage independently of macrolide activity. In animal models, treatment with clarithromycin specifically prevented adverse pregnancy and neonatal outcomes induced by *Ureaplasma parvum* intra-amniotic infection, supporting its continued inclusion in empiric regimens when susceptibility is unknown (140). Second, in settings where molecular susceptibility testing is feasible, resistance-guided sequential approaches, such as an initial doxycycline course followed by azithromycin or moxifloxacin according to macrolide resistance genotype, substantially improve treatment outcomes for infections attributable to macrolide-resistant strains (141). It is important to note that both doxycycline and fluoroquinolones, the standard second-line agents for *M. genitalium*, are generally avoided in pregnancy due to concerns about fetal cartilage damage and potential teratogenicity. Therefore, antibiotic options for macrolide-resistant genital mycoplasma intra-amniotic infection remain limited and represent an important unresolved clinical problem.

## CURRENT CHALLENGES IN THE DIAGNOSIS OF INTRA-AMNIOTIC INFECTION

**Fig 8:**
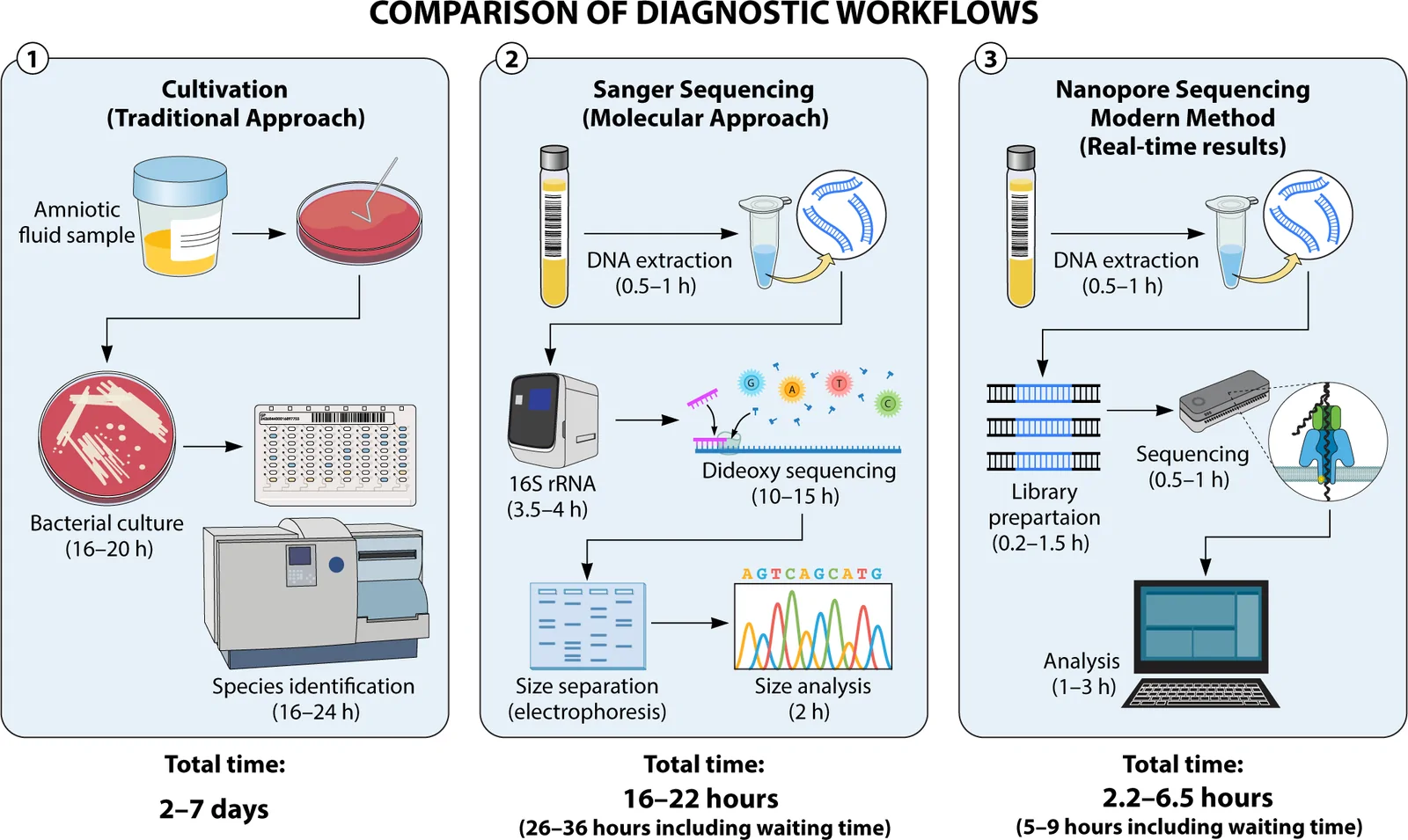
Comparisons of diagnostic methods for intra-amniotic infection. 16s rRNA, 16s ribosomal RNA.

The gold standard for bacterial identification is based on culture and biochemical phenotype identification. However, culture has several limitations, including the presence of significant unculturable species and the extremely time-consuming process required to obtain the results, which often spans several days and is not always in time for a clinical decision (Fig. 8) (142, 143). This delay has led to the clinical practice of initiating empirical antimicrobial agents before the precise identification of the microorganisms causing intra-amniotic infection. Indeed, in clinical obstetrics, empirical antimicrobial treatment is administered according to the clinical syndrome, with empirical intravenous ampicillin given for GBS prophylaxis in all patients with preterm labor and intact membranes (130). In patients with preterm pre-labor rupture of membranes, ampicillin with amoxicillin and erythromycin or azithromycin is given for the prolonged latency phase (144). In clinical chorioamnionitis, several antimicrobial regimens have been proposed (127). We believe that antimicrobial treatment should be targeted according to the amniotic fluid cavity status. To make it practical for clinical management, the ideal microbiological tool for diagnosing intra-amniotic infection should include rapid, accurate identification of pathogens; detection of antimicrobial resistance genes; and detection of polymicrobial infections. Given the shortcomings of the conventional identification method, sequence-based identification (also known as the “molecular sequencing-based technique”) should be adopted to overcome the barriers and improve performance in identifying unculturable species.

## LABORATORY METHODS USED FOR THE DIAGNOSIS OF INTRA-AMNIOTIC INFECTION

The detection of microorganisms in the amniotic cavity is currently based on three primary techniques: (i) direct microscopic examination; (ii) cultivation-based identification; and (iii) molecular identification. In this review, we will focus on such methods for microorganism identification.

### Direct microscopic examination

The initial step of bacterial identification from amniotic fluid is direct microscopic examination, including Gram staining for bacteria, acid-fast and modified acid-fast staining for higher bacteria, and potassium hydroxide preparation for fungi. Two main limitations constrain the clinical utility of Gram staining in the context of intra-amniotic infection.

1. The technique cannot detect bacteria that lack a rigid peptidoglycan cell wall. The most prevalent organisms in intra-amniotic infection, including *Ureaplasma* spp. and *Mycoplasma* spp., are phylogenetically wall-deficient and are entirely invisible to Gram staining regardless of their concentration in the specimen (145, 146).
2. The technique requires a threshold bacterial burden for reliable detection. Romero et al. (147) demonstrated that Gram stain sensitivity varies with bacterial concentration, reaching approximately 80% only when counts exceed 10^5^ CFU/mL. Concentration below 10^4^ cells/mL results in substantial deterioration in sensitivity, though cytocentrifugation of the specimen prior to staining can partially compensate (148). Against a culture reference standard, Gram staining of amniotic fluid achieves a sensitivity of approximately 64% and a specificity of 99% (Table 3). A negative Gram stain, therefore, provides meaningful reassurance but cannot exclude intra-amniotic infection, particularly when fastidious or cell wall-deficient organisms are clinically likely. The primary value of Gram staining in this setting is rapid preliminary identification of gram-positive cocci or gram-negative rods when bacterial burdens are sufficiently high.

**TABLE 3:** Diagnostic performance of bacterial detection methods compared to the cultivation method^*a*^

Method	Limit of detection	Sensitivity	Specificity	Positive predictive value	Negative predictive value	References
Gram stain	10,000 CFU/mL	64% (7/11)	99% (108/109)	88% (7/8)	96% (108/112)	(59)
Specific PCR (*Ureaplasma*/*Mycoplasma*)	100–1,000 genome copies	92% (12/13)	89% (101/113)	50% (12/24)	99% (101/102)	(149)
16S ribosomal RNA PCR/sequencing	10–100 genome copies	53% (10/19)	95% (277/293)	38% (10/26)	97% (277/286)	(150)
Metagenomic NGS (other body fluid specimens)	~100–10,000 genome equivalents	58% (134/231)	85% (117/137)	87% (134/154)	55% (117/214)	(151)

^*a*^ PCR, polymerase chain reaction; rRNA, ribosomal ribonucleic acid; and CFU, colony-forming unit.

### Cultivation-based identification

Cultivation-based identification methods remain a widely used, or gold-standard, tool for detecting pathogenic bacterial species in most clinical laboratories (146). Microbiological cultivation uses nutrient-rich media (i.e., blood agar and tryptic soy agar), allowing a wide range of bacteria to grow. Selective agar and differential agar are typically used to identify specific bacterial groups (152). MacConkey agar, for example, is both selective and differential: bile salts and crystal violet inhibit the growth of most gram-positive bacteria, permitting selective recovery of gram-negative organisms (selective), while the incorporation of lactose together with a pH indicator differentiates lactose-fermenting bacteria (differential), such as *Escherichia coli* and *Klebsiella* spp., which produce pink colonies, from non-lactose-fermenting bacteria, such as *Pseudomonas aeruginosa* and *Proteus* spp., which produce pale or colorless colonies. Once the bacterial strain is recovered, the colony is obtained for subsequent species identification, including biochemical testing or matrix-assisted laser desorption/ionization time-of-flight mass spectrometry (MALDI-ToF MS) (153). Importantly, the growing colonies can be further used for antimicrobial susceptibility testing and whole-genome sequencing.

#### Biochemical method

The growing bacterial colonies are collected for species identification based on their biochemical properties, including carbohydrate metabolism, movement, and enzyme production (146). Even though cultivation-based identification is currently used by several clinical microbiology units as a routine laboratory service, this technique requires well-trained, experienced technicians to interpret biochemical reactions of the bacterial culture. In addition, a large panel of biochemical tests is necessary for accurate bacterial species identification (146).

#### Matrix-assisted laser desorption-ionization-time-of-flight mass spectrometry method

**Fig 9:**
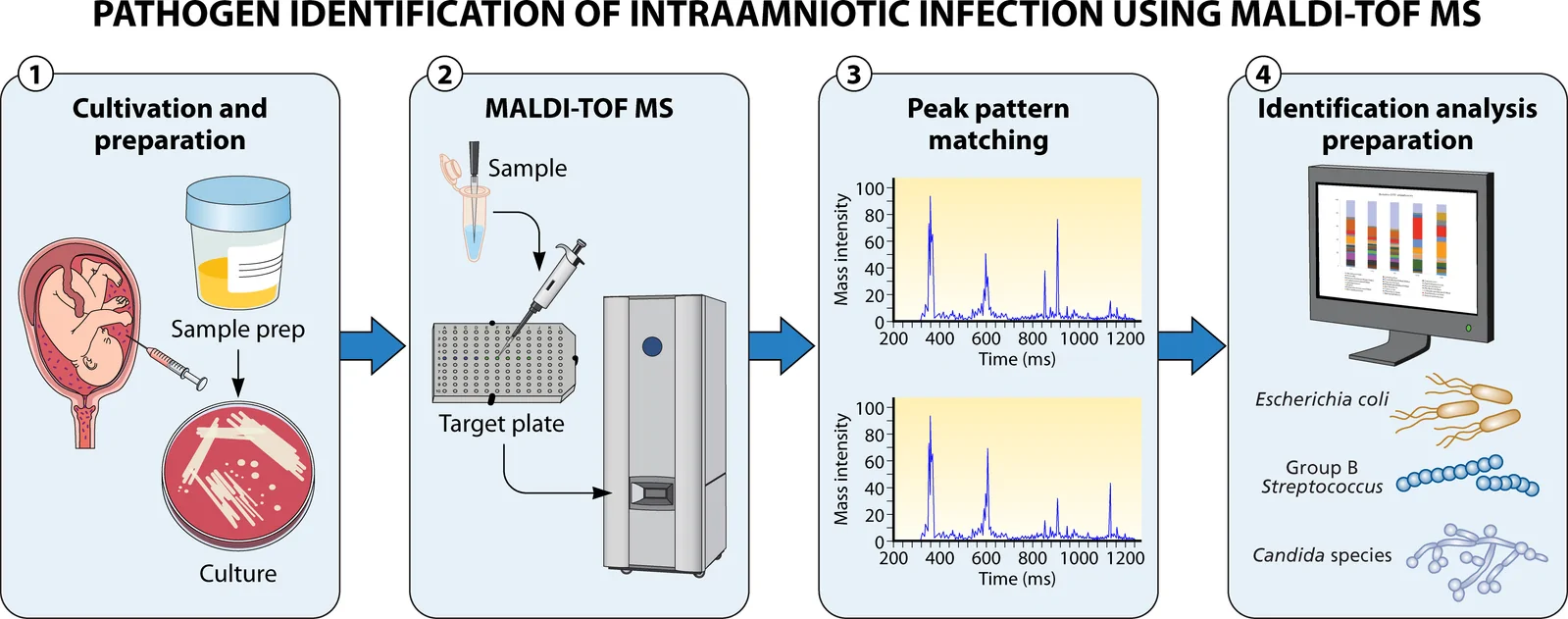
Workflow of pathogen identification using matrix-assisted laser desorption/ionization time-of-flight mass spectrometry.

MALDI-ToF MS has become a game changer in bacterial identification since its introduction to clinical microbiology (154). The principle of the MALDI-ToF identification method is to create a mass spectrometry profile called a peptide mass fingerprint (PMF) based on the detection of ionized peptide molecules and the duration that molecules use to reach the detector (time-of-flight). Identification of the causative pathogen is performed by matching the queried PMF to the MS profile in the database. This identification method can yield results within a few minutes of introducing the colony into the protein extraction process (154). The major limitation of this technique is that it can be used only if the microorganism can be cultured. Additionally, an up-to-date MS database is required for PMF matching (Fig. 9).

### Molecular microbiological technique

Molecular sequencing-based microbial identification has enabled us to enhance the accuracy of infection diagnosis by improving the identification of non-culturable and fastidious microorganisms (155–158). In most clinical microbiology units, polymerase chain reaction is used to identify known pathogens. PCR enables pathogen identification by binding to the genetic content (template) and amplifying the target. In conventional PCR, the PCR product is visualized by gel electrophoresis to determine whether the target (pathogen) is present (159). In clinical medicine, the 16S ribosomal RNA (16S rRNA) gene sequencing, a highly conserved genetic marker in bacteria, is frequently used to identify poorly characterized pathogens (156). Sequencing of the 16S rRNA gene, which decodes phylogenetically informative regions, can identify microbes across broad taxonomic levels, including previously uncharacterized species (155).

#### Targeted PCR

For targeted or specific PCR, primers are designed to bind to genetic sequences unique to a genus or species. For example, the *ure*B gene is the target for *Ureaplasma*-specific PCR (160). Targeted PCR has been used extensively for pathogen identification in reproductive tract infections, especially in vaginal discharge, given the suspected known pathogen panel (161, 162). In diagnosing intra-amniotic infection, specific PCR outperforms cultivation-based methods (149, 163). The main disadvantage of targeted PCR is that it is applicable primarily to well-studied pathogens and known bacteria.

Clinical validation of targeted PCR in intra-amniotic infection was demonstrated by a nested PCR-based assay that detects *Mycoplasma*, *Ureaplasma*, other bacteria, and fungi in amniotic fluid within 3 h of sample collection (164). Across 300 amniotic fluid samples, agreement between PCR and conventional culture was 93.3%, 94.3%, and 89.3% for *Mycoplasma*, *Ureaplasma*, and other bacteria, respectively. Moreover, targeted PCR extends directly into treatment monitoring. Yoon et al. used serial PCR-based *Ureaplasma* detection in combination with amniotic fluid culture to monitor the microbiological response to the triple antibiotic regimen (ceftriaxone, clarithromycin, and metronidazole) in patients with preterm labor and/or intra-amniotic (21) inflammation. This study underscores that targeted PCR not only is a diagnostic tool but also serves as a useful tool for microbiological confirmation of treatment response.

#### Broad-range PCR

Broad-range PCR provides greater resolution for bacterial identification. In principle, the target of broad-range PCR is conserved genetic sequences within the kingdom that can differentiate organisms across groups (from phylum to species). The 16S ribosomal RNA gene and the internal transcribed spacer (ITS) are generally used as PCR targets for broad-range approaches to bacteria and fungi, respectively (155). The presence of a broad-range PCR product must be followed by DNA sequencing to determine the pathogen’s species. 16S rRNA sequencing has been used to investigate the association between the vaginal microbiome and pregnancy outcome (165).

In intra-amniotic infection, 16S rRNA PCR is used to detect pathogenic bacteria to demonstrate microbial invasion of the amniotic cavity (58, 86, 87, 103). Previous studies demonstrated that broad-range 16S rRNA PCR improved the detection rate of bacteria from 34% (culture alone) to 45% (PCR alone) and 50% (culture and PCR combined) in amniotic fluid for the diagnosis of intra-amniotic infection (11). Moreover, a positive result of broad-range 16S rRNA PCR in amniotic fluid in patients with preterm labor is associated with acute histologic chorioamnionitis, acute funisitis, and preterm delivery, implying the presence of true intra-amniotic infection (10).

#### Whole-genome sequencing

Whole-genome sequencing provides the complete DNA sequences of a microorganism (166). In comparison to other identification methods, WGS enables precise taxonomic classification, leading to the discovery of novel species (167). The approach provides essential information, including antibiotic resistance and virulence factor-associated genes, allowing for targeted antimicrobial administration.

The advantages of WGS for pathogen identification include precise bacterial species identification, classification, and associated information, such as antibiotic resistance and virulence genes. However, this method has the drawback of requiring colonies obtained through cultivation. Through the use of whole-genome hybrid sequencing to characterize a strain of *Streptococcus oralis* recovered from the amniotic fluid, vaginal swab, and fetal blood of a patient presenting with pre-labor rupture of membranes and intra-amniotic infection, genomic analysis revealed that this strain harbored the macrolide resistance gene *erm*B, the tetracycline resistance gene *tet*(M), mutations in penicillin-binding proteins, and a repertoire of predicted virulence factors (168). Initially, *Streptococcus mitis* was identified by conventional microbiological identification (cultivation or 16S rRNA PCR). This study highlights a critical limitation of conventional diagnostics: pathogens carrying clinically significant resistance profiles may be misidentified or undercharacterized without genomic resolution, leading to suboptimal empiric therapy. Moreover, whole-genome hybrid sequencing of *Enterococcus faecium strain* RAOG174, which belonged to ST17 (a lineage associated with clinical antibiotic resistance and nosocomial infections), demonstrated the presence of vancomycin resistance genes, aminoglycoside resistance determinants, and mobile genetic elements, including bacteriophage sequences, providing a comprehensive genetic basis for the resistance phenotype that would not have been detectable by standard disc diffusion susceptibility testing alone (169). It could be proposed that genomic-level characterization is essential for accurate pathogen identification and appropriate antibiotic selection in intra-amniotic infection.

**Fig 10:**
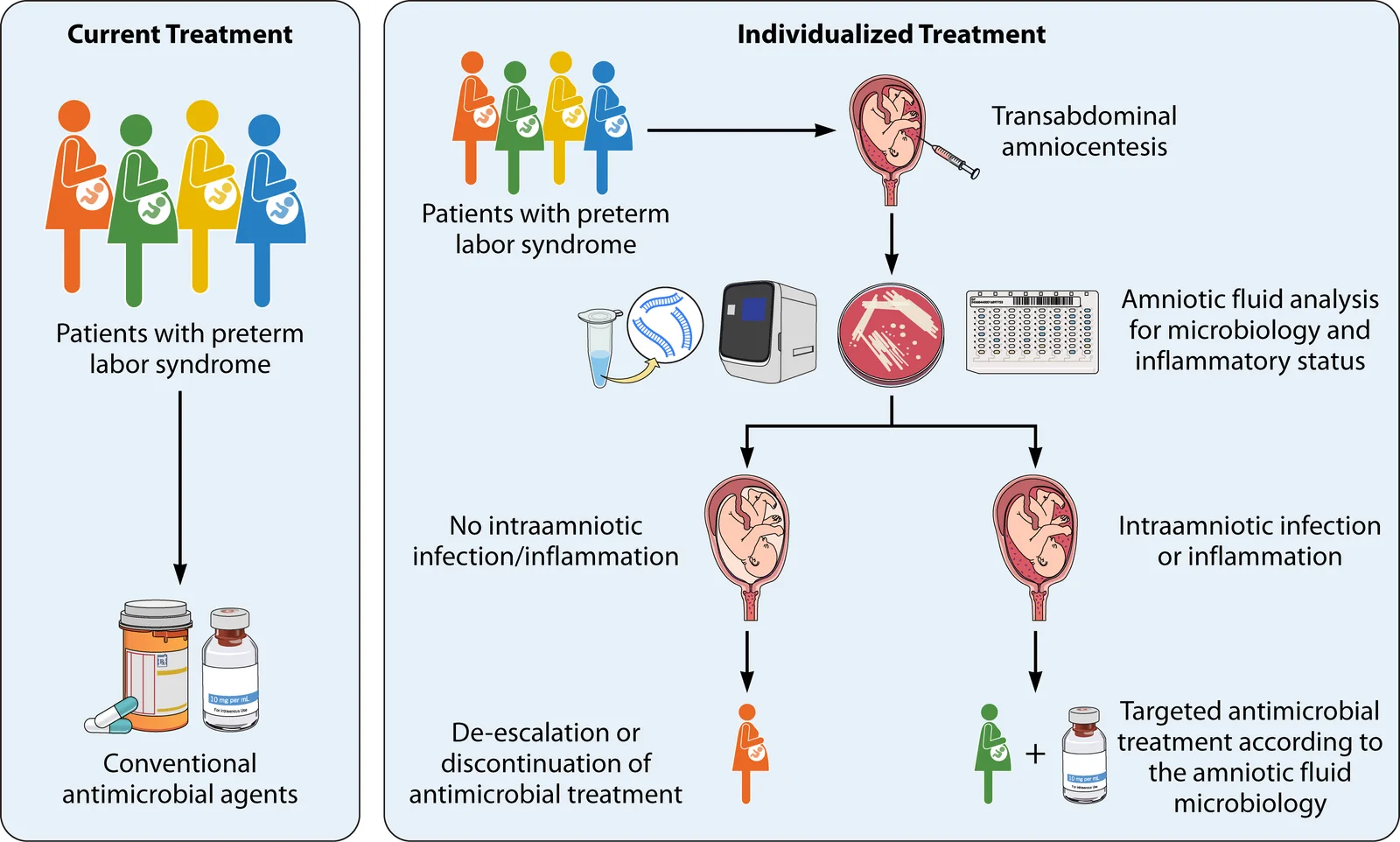
Proposed conceptual framework of individualized antimicrobial treatment according to amniotic fluid microbiology. The current or conventional treatment (left) and the individualized treatment according to the amniotic fluid microbiology result (right).

Recently, a new DNA WGS sequencing feature, called “adaptive sampling,” has been used directly from the specimen (amniotic fluid) rather than culture for rapid diagnosis of intra-amniotic infection (53). Chaemsaithong et al. (53) demonstrated that nanopore adaptive sampling of pus in amniotic fluid enables direct detection of bacteria and their antimicrobial resistance genes in intra-amniotic infections without prior cultivation. Specifically, using nanopore adaptive sampling, reads could be mapped to Prevotella species after 15 min of sequencing. Accurate genome sequencing was obtained after 12 h of sequencing, with the *Prevotella bivia* genome, which contained ceftriaxone or β-lactamase antimicrobial resistance genes (53). The *Prevotella bivia* could not be recovered by the cultivation technique. Information on antimicrobial resistance is key to achieving individualized antibiotic treatment rather than broad-spectrum antibiotics, which may not be effective against the pathogen causing intra-amniotic infection. Empiric regimens should, therefore, be guided by local antimicrobial resistance surveillance data, and targeted, genomically informed therapy should be implemented as rapidly as microbiological data allow. Figure 10 illustrates the conceptual framework for individualized treatment of intra-amniotic infection based on amniotic fluid microbiology and antimicrobial resistance genes.

#### Metagenomic sequencing

Metagenomic next-generation sequencing (mNGS) is an emerging tool that enables microbial identification directly from specimens without the need for cultivation. The core principle of mNGS is to detect the sequence of genetic material of any organism in the sample of interest. Therefore, mNGS has been widely used to prove culture-negative infections (143, 170–172). mNGS not only detects species but also identifies virulence and antimicrobial resistance genes (173). For example, mNGS can identify pathogens and their antibiotic susceptibility in complicated urinary tract infections with accuracies of 99% and 90%, respectively (174). Therefore, mNGS has transformed the diagnostic pillar of infectious diseases by enabling robust pathogen identification, thereby enabling more precise clinical decision-making and management (175). In an amniotic fluid study, mNGS increased the detection rate of microorganisms in the amniotic cavity from 2.5% (culture) to 17.5% (mNGS) in patients presenting with preterm labor (176). In another study, high-throughput sequencing of cell-free DNA with the use of a bioinformatics noise filtering tool in amniotic fluid was shown to be able to identify intra-amniotic infection and confirm the sterility of the amniotic cavity during normal pregnancy (7). Yet, the challenges of mNGS include distinguishing true pathogens from background microorganisms, especially in low-biomass samples such as amniotic fluid samples, which are more susceptible to contamination from reagents, non-sterile procedures, and the laboratory environment. Bioinformatic analysis, together with proper controls, including environmental and reagent controls, can establish a cutoff value for pathogen-background separation (7, 53, 177). To date, there is no standardized or well-established protocol for mNGS in low-biomass samples. Therefore, it is difficult to determine whether the detected microorganisms are pathogens or contaminants. A systematic review and meta-analysis of mNGS across infectious disease applications reported pooled sensitivity and specificity of 75% and 68%, respectively, with an area under the receiver-operating curve of 0.85, which is better than culture in sensitivity but lower in specificity, confirming that false-positive results from contamination or background microbiome sequences are a main concern in clinical practice (178). A positive mNGS result in amniotic fluid must therefore be interpreted in the context of clinical diagnosis or factors, parallel inflammatory biomarkers, and internal controls before being used to guide antibiotic initiation or modification.

The economic and infrastructural requirements of mNGS further constrain global availability. The cost per sample for shotgun mNGS, including library preparation, sequencing, and bioinformatic analysis, typically ranges from USD 200 to over USD 1,000 depending on platform and sequencing depth, placing it beyond routine reach in most low- and middle-income settings where intra-amniotic infection is most prevalent (179). Targeted next-generation sequencing approaches, which sequence a defined panel of pathogen-specific regions rather than the entire metagenome, offer substantially reduced cost (approximately 25%–50% of full mNGS), shorter turnaround times, and lower host DNA interference and are emerging as a practical intermediate between targeted PCR and full metagenomics (180). Until standardized mNGS protocols for low-biomass sterile-site specimens are validated and commercially scaled, clinical implementation should remain restricted to settings with dedicated bioinformatic support and quality-controlled contamination monitoring.

A separate discussion of cell-free DNA (cfDNA) sequencing in pregnancy is warranted in this context. The application of cfDNA in pregnancy has been widely used, mostly in the diagnosis of genetic diseases (181). Moreover, a cell-free transcriptomic study reveals more insight into fetal development (182). Microbial cfDNA sequencing has been explored as a non-invasive approach for detecting infectious pathogens in peripheral blood without amniocentesis. In immunocompromised patients, microbial cfDNA sequencing from peripheral blood demonstrated positive agreement with conventional diagnostic methods in 70% of cases and identified probable pathogens in culture-negative sepsis cases (183). Whether this technology can reliably detect the low-burden infections characteristic of intra-amniotic infection from non-invasive maternal blood sampling remains an open question. Kacerovsky et al. (184) demonstrated that levels of cell-free host DNA, including nuclear and mitochondrial DNA, were associated with the presence of intra-amniotic infection and microbial invasion of the amniotic cavity. Given the placenta’s role as a selective barrier and the typically low absolute concentrations of microbial DNA in early intra-amniotic infection, the sensitivity of blood-based microbial cfDNA sequencing for the diagnosis of intra-amniotic infection is likely to be lower than that of amniocentesis, and a prospective study and clinical validation are required before deployment.

Future research is needed to develop an appropriate mNGS protocol for diagnosing intraamniotic infection. Tables 3 and 4 and Figure 8 summarize molecular approaches used in pathogen identification.

**TABLE 4:** Comparison of molecular methods for the identification of pathogens^*a*^

Diagnostic approach	Targeted PCR (singleplex/ multiple)	Broad-range targeted PCR (16S rRNA gene or ITS) sequencing	Shotgun metagenomic NGS	Whole-genome sequencing
Spectrum of detection	Limited to selected pathogens	Bacteria only (for 16S) Fungi only (for ITS)	Bacteria, viruses, fungi, and parasites	Single live organism from culture or a direct specimen
Ability to identify unknown pathogen	None	High	Very high (hypothesis-free)	High (cultured organisms or host depleting step if direct specimen)
Taxonomic resolution	Species/strain (if target-specific)	Genus to species (variable)	Species to strain (variable)	Species, strain, single nucleotide polymorphism level
Turnaround time	Hours	1–3 days	1–7 days	Days to weeks
Key strengths	Rapid, high sensitivity, and clinically actionable	Detects unculturable or fastidious bacteria (16S); sensitive fungal identification (ITS)	Comprehensive pathogen discovery	Identification of antimicrobial resistance genes, virulence factors, and epidemiology information
Major limitations	Cannot detect unexpected organisms	Cannot detect viruses; limited to bacteria (16S) and fungi (ITS); database dependence	Host DNA background and interpretation challenges	Requires culture, cost, and expertise
Typical clinical application	Known syndromes or suspected causes (tuberculosis, sexually transmitted diseases, and viral infections)	Culture-negative sterile-site infections or invasive fungal infections	Unknown etiology infections	Outbreaks, antimicrobial resistance gene profiles, and surveillance
References	(185)	(186, 187)	(185, 188)	(53, 189, 190)

^*a*^ PCR, polymerase chain reaction; ITS, internal transcribed spacer, which is a genetic region used in molecular methods like PCR and sequencing to identify and classify pathogens (disease-causing microbes) by targeting specific DNA sequences; and rRNA, ribosomal ribonucleic acid.

## DNA SEQUENCING PLATFORM

DNA sequencing is an important tool for improving identification resolution, especially for poorly identified microorganisms by cultivation-based techniques and for culture-negative clinical specimens. Currently, there are three sequencing technologies commonly used in clinical microbiology: (i) Sanger sequencing; (ii) short-read sequencing; and (iii) long-read real-time sequencing.

### Sanger sequencing

The Sanger sequencing method, one of the earliest DNA sequencing techniques, remains a common sequencing method for routine microbiological identification in clinical medicine. This sequencing method, invented by Frederick Sanger in the 1970s, is based on chain termination using dideoxynucleotide triphosphates (191). Sanger sequencing is generally used for species determination, following broad-range PCR, such as full-length 16S rRNA sequencing, due to its accuracy and time efficiency. However, in polymicrobial specimens, the presence of multiple templates yields uninterpretable Sanger reads (192). In intra-amniotic infection, 16S rRNA gene sequencing via the Sanger method unveils microorganisms, such as *Ureaplasma* and *Mycoplasma*, as causative agents of intra-amniotic infection (10, 193), and this method has been used clinically for the diagnosis of intra-amniotic infection (67, 83–85, 128, 194–196).

### Short-read sequencing

The short-read or sequencing-by-synthesis DNA sequencing technique is characterized by high throughput and accuracy. Common characteristics of the short-read sequencing method include preparing short DNA sequences (a library) from genomic DNA by fragmentation and amplification. Then, numerous short (150–300 base pairs) DNA sequences are synthesized in parallel. Multiple short DNA sequences (reads) can be obtained for downstream analyses, including gene identification or genome assembly (191).

Because this sequencing method generates multiple nucleotide sequences, it can identify multiple bacterial species from polymicrobial specimens. A study by Romero et al. (103) demonstrated that short-read 16S rRNA sequencing covering the V3–V4 region could detect unculturable bacteria in amniotic fluid samples. However, taxonomic identification was limited to the genus level due to the short read length, which covers the partial length of the 16S rRNA gene (197). To overcome the limitations of first- and second-generation sequencing, long-read sequencing technology has played a significant role in species identification, providing long reads that span nearly the full length of the 16S rRNA gene (198).

### Long-read sequencing

Long-read sequencing enables higher-resolution DNA analysis by increasing the read length, yielding a full sequence of the target gene. Oxford Nanopore technology (ONT), one of the long-read sequencing platforms, enables real-time analysis, accelerating analysis time. Briefly, the principle of nanopore sequencing is that a single DNA strand passes through a nanoscale pore, altering the ionic current as it moves (199–202). A change in ionic current is detected and encoded into DNA sequences (Table 4 and Fig. 8) (203). Similar to short-read sequencing, processing and interpreting data from ONT require bioinformatic analysis (204). Several software packages from both ONT and third parties have been developed to improve the ability of ONT (204). Long-read sequencing provides a significant advantage over short-read technology in that it can decode the human genome with better resolution and shorter duration (205). In clinical medicine, long-read sequencing has enhanced the diagnosis of genetic diseases (206, 207) and prenatal genetic screening (208).

In diagnosing intra-amniotic infection, recent studies have demonstrated that 16S rRNA nanopore sequencing can provide species identification results within approximately 10 h of DNA extraction, compared with at least a few days for conventional cultivation or 16S rRNA Sanger sequencing (86, 87). This shortened reporting time enables clinicians to make informed clinical management decisions promptly (87). The 16S rRNA nanopore sequencing method has 88.9% sensitivity, 95.4% specificity, and a positive likelihood ratio of 19.1 for diagnosing intra-amniotic infection in patients with preterm labor and preterm pre-labor rupture of membranes (87). Despite the robustness of the method, the limitations of 16S nanopore sequencing for the diagnosis of intra-amniotic infection include limited availability of bioinformatic infrastructure, high sequencing costs, and a high risk of contamination, requiring a negative control and a proper cutoff for bacterial reads to detect true pathogens.

## THE ROLE OF RAPID DIAGNOSIS OF INTRA-AMNIOTIC INFECTION IN NEONATAL MANAGEMENT

Intra-amniotic infection is a significant maternal risk factor for early-onset neonatal sepsis, with the risk increased approximately fivefold relative to unexposed neonates (209). Rapid and accurate diagnosis of intra-amniotic infection benefits the neonate in two main aspects. First, when the causative pathogen and its antibiotic susceptibility are known before or at delivery, neonatal empiric therapy can be rationalized rather than initiated solely on the basis of maternal clinical signs, which is ineffective in diagnosing neonatal septicemia. Second, when amniocentesis-based microbiological assessment confirms the absence of intra-amniotic infection, neonatal antibiotic exposure can be safely curtailed or avoided. The use of the 16S nanopore sequencing method described above had a turnaround time of approximately 10 h from DNA extraction, exemplifying the potential of rapid molecular diagnostics to enable microbiologically informed decision-making within a clinically relevant timeframe for patient management for both mothers and neonates (86, 87).

The most immediate clinical benefit of rapid intra-amniotic infection diagnostics is supporting antibiotic stewardship by enabling earlier de-escalation of antibiotics in intra-amniotic infection-exposed neonates at low infection risk and by ensuring rationalized therapy in those with true infection, including infections caused by organisms resistant to standard empiric regimens. Integration of rapid maternal microbiological data into standardized neonatal risk-stratification frameworks represents an important translational step toward improving neonatal outcomes in infection-associated preterm birth.

## FUTURE PERSPECTIVE

The direction of microbiological diagnosis for intra-amniotic infection has shifted from culture-based methods toward culture-independent molecular approaches. Real-time metagenomic sequencing represents an ideal future platform because it enables unbiased detection of bacterial, viral, and fungal pathogens without prior assumptions and allows characterization of polymicrobial infections. However, amniotic fluid is a low-biomass specimen in which microbial DNA is often present at extremely low concentrations relative to host nucleic acids, making results highly susceptible to background contamination and analytical noise. Therefore, robust diagnostic strategies must incorporate strict contamination control, host DNA depletion or microbial enrichment, and advanced bioinformatic algorithms for noise screening. Integration of functional genomic analysis for antimicrobial resistance and virulence, together with host-response biomarkers, may further distinguish true infection from sterile inflammation. An automated, standardized, and rapid molecular diagnostic framework could provide robust, clinically actionable results for intra-amniotic infection and other low-biomass-associated infections. In addition, the development of a non-invasive method for diagnosing intra-amniotic infection is a frontier (42, 210).

## CONCLUSIONS

Intra-amniotic infection remains a significant threat to maternal and fetal health worldwide. Although several conventional clinical microbiological techniques are routinely employed for pathogen identification, these methods are often limited by prolonged turnaround time and their inability to provide comprehensive information on antimicrobial resistance determinants. In contrast, molecular approaches, including targeted gene sequencing, whole-genome sequencing, and metagenomic sequencing, substantially improve the resolution and accuracy of pathogen detection, particularly for fastidious and unculturable microorganisms, and enable more precise species-level identification as well as characterization of antimicrobial resistance genes. However, the selection of an appropriate molecular method should be guided by practical feasibility in both clinical and research settings, considering factors such as cost, availability of laboratory infrastructure, technical expertise, and time to result. Targeted sequencing approaches are advantageous for rapid and economical detection of known pathogens, whereas whole-genome sequencing is better suited for in-depth genomic characterization of cultured isolates, including resistance and virulence profiling. Metagenomic sequencing provides the most comprehensive assessment of microbial communities and is especially valuable for polymicrobial or culture-negative infections, but its broader implementation remains limited by higher financial costs and analytical complexity. Therefore, an integrated and context-dependent strategy that balances diagnostic performance with logistical and economic feasibility is essential for optimizing the investigation and management of intra-amniotic infection.

## Data Availability

All data generated or analyzed are included in this published article. For further information, please contact the corresponding authors.

## EDITOR'S NOTE

**Ed. Note:** A potential conflict of interest was identified after acceptance of this paper, and a CMR editor conducted an additional review to ensure the integrity and fairness of the peer review process.
